# Mass intrahospital transport of the neonatal intensive care unit: a systems-based approach to safety, efficiency, and family-centered care

**DOI:** 10.1038/s41372-026-02721-x

**Published:** 2026-05-27

**Authors:** Pankaj Sakhuja, Naveed Durrani, Dhullipala Anand, Nqobile Sigola, Danica Causing, Ernestine Yangamen Kalla Lottin, Ali Alqudah, Hassan Deni, Haytham Ali, Duane Wong, Susanne Bohm-Langford, Samir Gupta

**Affiliations:** 1 Division of Neonatology, Department of Paediatrics, Sidra Medicine, Doha, Qatar; 2 Weill Cornell Medicine, Doha, Qatar; 3 Clinical Engineering, Sidra Medicine, Doha, Qatar; 4 Allied Health Department, Sidra Medicine, Doha, Qatar; 5 Clinical Operations, Sidra Medicine, Doha, Qatar; 6 Department of Engineering, Durham University, Durham, UK

**Keywords:** Paediatrics, Health services

## Abstract

**Objective:**

To design and evaluate a systems-based approach for safe, efficient, and family-centered mass intrahospital transport (M-IHT) of critically ill neonates during two planned NICU relocations.

**Methods:**

This prospective QI initiative followed SQUIRE 2.0 guidelines. Work system design was guided by systems-based patient safety frameworks, Operations/Management Science principles, integrating context evaluation with proactive, multidisciplinary, simulation-based planning to mitigate risk and ensure real-time adaptability. Outcomes were evaluated using prospectively collected multi-source data.

**Results:**

Both relocations were completed without clinical complications, equipment failures, or service disruption. Staff surveys showed >90% positive responses across domains. Parents reported high satisfaction, perceived safety, and effective communication. Direct observations confirmed smooth coordination, role clarity, workflow adherence, and real-time responsiveness. The work system was successfully reapplied with consistent performance and improved efficiency.

**Conclusions:**

M-IHT of critically ill infants was conducted safely and efficiently using a reliable, adaptable, and sustainable system-based framework offering transferable lessons for complex operations.

## Introduction

Intrahospital transport (IHT) can disrupt physiological stability, with adverse events (AEs) reported in approximately 25% of transported neonates and rates ranging from 0.1% to 69% across all age groups. While clinical instability, equipment issues, and communication gaps are well documented [1–4], parental well-being, operational efficiency, safety climate, and team dynamics also contribute to transport-related risks [5, 6].

Many errors in healthcare arise from poorly designed work systems. Human factors engineering focuses on optimizing interactions among system components, including people, tasks, tools, and environments [7, 8]. A systems-thinking approach emphasizes the interconnectedness of these components and how their interactions influence outcomes. System-based frameworks such as the Systems Engineering Initiative for Patient Safety (SEIPS) and tools like the Intrahospital Transport Safety Scale (IHTSS) can help identify and mitigate risks during IHT [9–12].

Mass intrahospital transport (M-IHT) may be required during planned NICU renovations, amplifying IHT-related risks, necessitating meticulous planning to prevent AEs. The literature on M-IHT remains sparse, and challenges related to teamwork, workflows, and communication remain underexplored [13].

A planned NICU renovation provided an opportunity to design and test a work system for M-IHT of high-acuity neonates, prioritizing safety, operational efficiency, and family experience. This initiative was guided by resilience science principles, emphasizing anticipation and proactive risk management, and aligned with SQUIRE 2.0 aims of theory-informed planning, contextual evaluation, and systematic assessment of outcomes [14, 15]. We report the system-level planning, execution, and evaluation of this M-IHT, offering a transferable model for other units planning large-scale IHT.

## Methods

### Setting and stakeholders

This systems-based initiative was undertaken to address a service need arising from the planned renovation of a 24-bed intensive and high-dependency care area within a 42-bed quaternary NICU at Sidra Medicine, Doha, Qatar. The unit spans across two floors and is designed with single-family rooms (SFR) to support family-centered, specialized, multidisciplinary care for critically ill neonates, including those requiring cardiac surgery. The 24-bed intensive and high-dependency care area (Unit 3C) on the third floor was relocated for renovation (Relocation 1) to a temporary area (Unit 3A) on the same floor and subsequently returned to the renovated Unit 3C one year later (Relocation 2). Each unit has two entry doors; the transport distances between the two units range between 120 and 140 meters, depending on the doors used (confirmed by pedometer measurements and architectural plans).

Stakeholders included NICU staff (nurses, allied health professionals, and physicians), neonates, their parents as partners in family-centered care, and the hospital’s administrative, management, and technical teams (biomedical engineering, information technology (IT), facilities, portering, and security services).

### Context assessment

Several environmental and operational factors, including single-family room layout, high-acuity case mix, route, and the challenges of transporting critically ill neonates, were examined through multidisciplinary team (MDT) discussions, real-time observations, and simulations. Planning also considered infrastructure readiness (e.g., staged room availability due to limited monitors), infection control, staffing and equipment, continuity of care, and parental needs and expectations. Simulations tested transport routes, teamwork, equipment and resource needs, and workflows across different patient acuities. The full transport cycle (from pre-transport preparation to team return) took approximately 30–40 min for high-acuity neonates (ventilated, on multiple inotropes, inhaled nitric oxide), 20–30 min for moderate-acuity neonates (stable ventilated), and 15–20 min for low-acuity neonates (stable on non-invasive or no ventilation). These insights guided staffing, resource coordination, contingency planning, time allocation, and transfer sequencing. A 30-min interval was planned between consecutive transfers to ensure smooth flow and minimize corridor congestion. The sickest infants, at higher risk of instability, were scheduled later among the ventilated cohort to avoid disrupting the relocation process. Neonates receiving high-frequency oscillatory ventilation (HFOV) were transported using their own ventilator to minimize the risk of destabilization.

### Work system design

**Fig. 1:**
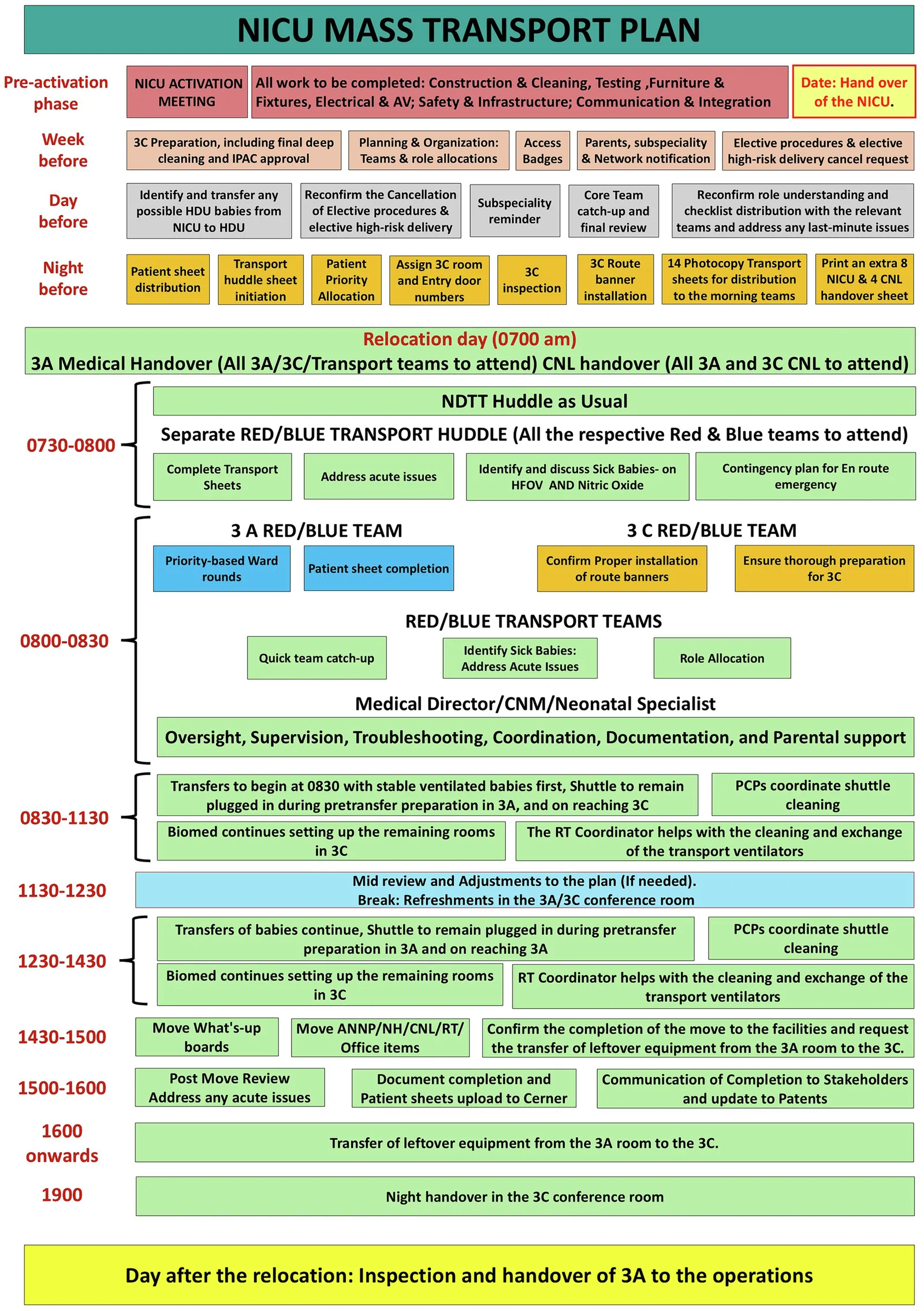
Operational plan for Relocation 2 (Temporary NICU 3 A → Renovated NICU 3 C), showing five sequential operational phases: Pre-activation, Week Before, Day Before, Night Before, Move Day, and Post-move. Red and Blue Transport teams worked in parallel with their respective NICU unit teams under real-time leadership oversight. Abbreviations: CNL Clinical Nurse Leader, PCP Patient Care Partner, RT Respiratory Therapist, HFOV high-frequency oscillatory Ventilation, NDTT Neonatal Delivery and Transport Team, IPAC Infection Prevention and Control, ANNP advanced neonatal nurse practitioner, CNM clinical nurse manager.

Our SEIPS-based work system (Table 1) comprised five domains—Organization and Teamwork, Environment, Tools and Technologies, Transport-related Tasks, and Communication [9, 10]. The IHTSS provided a detailed breakdown of these domains [11, 12], which were further refined using operations research (OR)/management science (MS) principles [16] and Safety Attitudes Questionnaire (SAQ) elements [17]. The comprehensive *“NICU Mass Transport Plan”* (Fig. 1), a key operational document outlining the phased workflow and overall strategy, was activated one week before the relocation. A proactive approach, in line with resilience science and SQUIRE 2.0 principles [14, 15], led to the development of detailed relocation and role-specific checklists, huddle sheets, patient data sheets, a workforce and equipment-management plan, and other operational tools. Communication was central, with key strategies including structured NICU staff briefings, regular family updates, and advance notification to national neonatal transport services to defer non-urgent neonatal transfers. Elective deliveries and procedures were rescheduled to prioritize NICU resources, and close collaboration with allied-health and support departments (biomedical engineering, facilities, IT, pharmacy/Omnicell, portering, and security) ensured infrastructure and equipment readiness. The Vocera call and code system supported continuous and urgent communication. Real-time coordination was led by neonatal attendings, clinical nurse leaders (CNLs), and zone-based neonatal specialists.

**Table 1:** System-based framework for guiding mass neonatal intrahospital transport, integrating key safety and systems-engineering tools.

WORK SYSTEM (A System-Based framework)		
Domains	Operational Tools (“Doing” & “Studying”)	Guiding Principles
**Organization and Teamwork**	**1. The NICU MASS Transport Plan** (Fig. 1) **2**. **Relocation Checklist** (Supplementary Appendix S1) **3**. **Workforce and Equipment Management Plan** (Supplementary Appendix S2): Defines team compositions and workflows across transport, primary, and receiving units (Red/Blue teams), including designated zone specialists, RT coordinators, and PCPs. It outlines the leadership structure, role-specific documentation, equipment logistics, and integration of Support Services (Biomed/facilities/security/portering) for environment preparation and standby support. **4**. **Transport Huddle & Patient data sheets** (Supplementary Appendix S3 and 4) **5**. **Staff survey and Patient survey**	**Systems thinking:** Ensured all people, tasks, tools, and environment worked in coordination and adapted efficiently to any change. **IHTSS**: Guided team structure, task-based workflows, environmental preparation, equipment management, and leadership support, enabling meticulous planning across all domains. **OR/MS:** Supported resource allocation through constraint management—optimizing team composition, equipment flow, transport sequencing, route planning, parental involvement, and contingency planning. **SAQ:** Informed teamwork climate, leadership visibility, and workload pacing through structured workflows and clear roles. Reinforced environmental and equipment readiness, staff morale, safety culture and consistent communication. **Resilience Science:** Ensured flexibility and responsiveness—enabling anticipation, learning from simulation, continuous monitoring, real-time troubleshooting, and built-in capacity for adjustment and recovery.
**Environmental Preparation**	**1**. **Simulation and Real-Time Observations** **2**. **NICU Preparation and Room Activation:** Clinical workspaces, nursing hubs, and IT stations were prepared in advance with staff access cards, and electrical/IT/biomed and infection-control readiness ensured. Rooms were prepared stepwise based on patient priority and transport order; only a few rooms were ready initially, with monitors freed from vacated rooms and reused to prepare the next rooms. **3**. **Transport Route Planning** (Supplementary Appendix S5)**:** Routes between the two NICUs were preassigned for each baby, with the directional signage and room-number banners.	
**Tools and Technologies:** **(Equipment Management)**	**1. Emergency kits and carts** **2**. **Transport Ventilators and Shuttles** **3. Battery and Gas Management** **4**. **Equipment Cleaning and Reuse Workflow**	
**Transport-Related Tasks**	**1**. **Patient Data Sheets:** Initiated by the bedside nurses the night before the relocation and were uploaded into the patient’s electronic record after the relocation. **2**. **Transport Huddle Sheets** **3**. **Priority Numbers:** Assigned the night before based on clinical stability and transport complexity. **4**. **Estimated Departure Times (EDTs):** EDTs were assigned to pace transfers, starting at 08:30 with an average interval of 30 min and the patient’s priority number. **5**. **Room allocation:** Twins were placed in adjacent rooms, and sicker infants were assigned larger rooms to accommodate more equipment **6**. **Pre-Transport Safety Checklists:** Standard NICU pre-transport checklists completed before transport. **7**. **Parent Involvement** **8**. **Planned Break and Debrief:** A midday review and staff break, and end-of-transfer debrief with refreshments.	
**Communication**	**1**. **Internal Team Communication and Coordination** **2. Communication with Hospital and External Stakeholders** **3. Communication with Families**	

### Study of the intervention(s)

We evaluated the impact of the work system using multiple data sources [18].

### Clinical outcome and operational measures

Clinical data were prospectively collected using standardized patient data sheets. These captured pre-, intra-, and post-transport vital signs, respiratory support, sedation, infusions, feeding status, any interventions, and care plan were documented during the primary care team ward rounds. AEs were defined as sustained physiological instability requiring intervention, unplanned clinical escalations, or equipment malfunctions. Routine oxygen titration to maintain target saturations was not classified as an AE. Transport times were categorized as:

- Total transport time: The duration of the entire relocation (from the departure of the first neonate to the arrival of the last).
- Individual transport time: The interval between departure and arrival for each neonate.

**Qualitative and survey measures:**

- Direct observations noted broader operational insights, including teamwork, role clarity, adherence to checklists, workflow efficiency, environmental and equipment readiness, and overall coordination.

- Staff survey guided by elements of IHTSS [11, 12] and SAQ [17] addressed all domains of the work system. It was distributed via the general NICU staff email list, with reminders sent only to staff directly involved and present during the relocation. Responses were reported on a four-point Likert scale (“from strongly disagree/very inadequate” to “strongly agree/very adequate”).
- Parental survey assessed communication, satisfaction, perceived safety, and continuity of care using a three-point scale (dissatisfied, satisfied, or very satisfied).

### Statistical analysis

Continuous variables were summarized as medians with interquartile ranges, and categorical variables as frequencies and percentages. Survey responses were presented as proportions across categories. Domain-level composite scores were generated by aggregating responses within each domain; scores of 3-4 were categorized as positive and 1-2 as negative. Open-text comments were analyzed thematically.

### Ethical considerations

The institutional review board (IRB) determined that this project did not constitute human-subject research. Clinical data were collected as part of standard care, and staff and parental surveys were voluntary and anonymous. No identifiable patient data was used. All methods were performed in accordance with the relevant guidelines and regulations.

### Use of generative AI

Generative AI (GPT-5; OpenAI, 2025) was used strictly for language clarity, consistency, and formatting. No scientific content, data analysis, or interpretation was generated by AI. All content was reviewed and approved by authors who take full responsibility for the accuracy and integrity of the work.

## Results

### Patient characteristics, safety, and efficiency outcomes (Tables 2 and 3)

Thirty-four neonates were transported across two relocations (17 per relocation). The most common diagnoses included complex congenital heart disease, necrotizing enterocolitis, and bronchopulmonary dysplasia, with detailed diagnostic sub-classification provided in Table 3. Documentation was incomplete for one infant in Relocation 1, resulting in 16 analyzed cases, while all 17 were analyzed in Relocation 2. There were more male infants in Relocation 2 (82% vs. 62%). Median postmenstrual age was similar between relocations; however, the median transport weight was lower in Relocation 2 (2420 g vs. 2700 g). A greater proportion of neonates required invasive ventilation, sedation, and >2 continuous infusions (47.0% vs. 31.2%) in Relocation 2.

There were no AEs or equipment malfunctions across either relocation. All infants maintained normothermia and stable vital signs throughout. Minor adjustments to inspired oxygen concentration were required in approximately one-third of neonates to maintain target saturations in both relocations; however, these did not meet the criteria for an adverse event.

The projected total transport time to relocate 17 neonates was 255 min, based on two transport teams working in parallel and an estimated 30 min per patient. Actual total transport times were shorter than estimated, with Relocation 2 completed in less time than Relocation 1 (197 vs. 225 min), although median individual transport times were slightly longer in Relocation 2 (6 vs. 4 min).

### Pre-transport events and operational insights

During Relocation 2, two neonates required additional pre-transport stabilization: one developed sepsis requiring intubation, while another became unstable during transition to the transport ventilator. Both were stabilized and transported without adverse events. These events necessitated adjustments to the planned transport sequence, disrupting staged room preparation and delaying readiness of the final room.

During Relocation 2, IT issues and non-functional electrical sockets were identified and managed effectively by pre-assigned standby teams (IT, facilities, and biomedical engineering).

Direct observations and informal feedback from frontline staff highlighted smooth coordination, clear role allocation, adherence to workflows and real-time responsiveness. Performance was consistent across both relocations, with improved efficiency observed during the reapplication of the work system in Relocation 2.

### Staff survey (Fig. 2)

A total of 57 responses were received for Relocation 1 and 61 for Relocation 2, with approximately 90% from staff directly involved. Positive scores exceeded 90% across all domains in Relocation 1 and more than 95% in Relocation 2. Feedback from Relocation 1 highlighted the need for earlier and broader communication, improved equipment and IT readiness, and additional nursing support. Most were addressed in Relocation 2; however, IT issues, minor facilities issues (e.g., bins, soap dispensers, electrical sockets), and requests for extra nursing support persisted. Staff consistently highlighted structured planning, visible leadership, teamwork, and communication, describing the process as “*well organized,” “safe*,” and *“excellently executed.”*

**Fig. 2:**
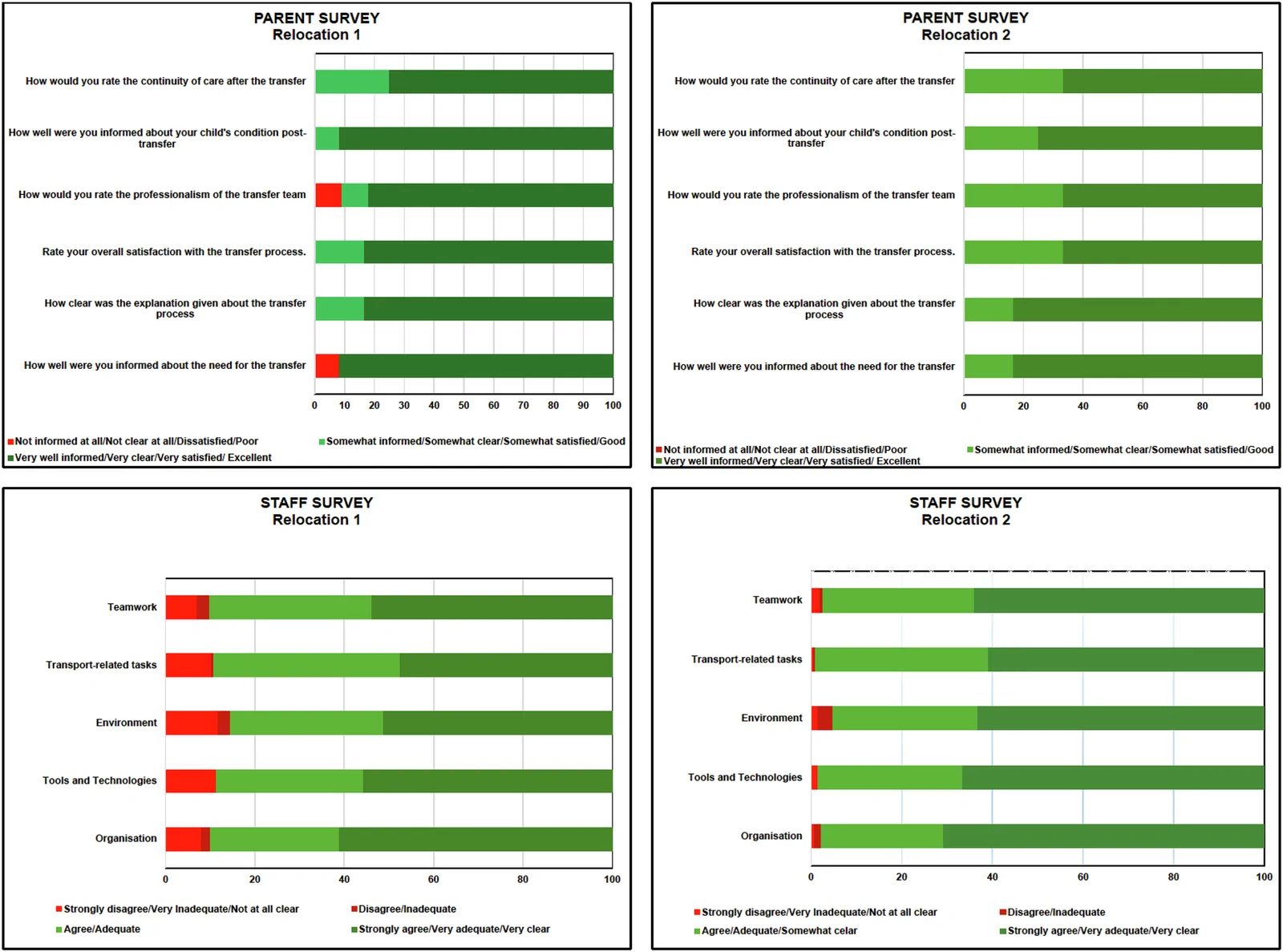
Parent and staff survey results following Relocation 1 and Relocation 2. Each bar represents composite domain scores, illustrating the distribution of responses from negative to positive categories.

### Parent survey (Fig. 2)

Twelve parents (70%) completed the survey following each relocation. All reported feeling well-informed and satisfied with the transfer and post-transfer care. One parent expressed concern about not being able to be present during the transfer in Relocation 1.

## Discussion

The M-IHT for high-acuity neonates was completed safely and efficiently, without clinical complications or service disruption. We achieved a high level of staff and parental satisfaction by applying a systems-thinking approach guided by the SEIPS framework [8–10]. Insights from the IHTSS [11, 12], resilience-science [15], OR/MS [16], and SAQ [17] helped operationalize the work system into practical, manageable components. Context evaluation identified modifiable factors (e.g., environmental preparation, workforce and equipment management, workflow coordination, and stakeholder communications) and non-modifiable constraints (e.g., patient acuity, unit layout, time constraints) [14, 17]. We anticipated these factors through simulation, direct observation, and team discussion, which enabled contingency planning and enhanced preparedness. The work system’s resilience was evident as unexpected clinical and operational disruptions were absorbed without compromising safety or efficiency [15]. The transported cohort reflected a broad range of diagnoses and clinical complexity, including a higher proportion of infants requiring advanced respiratory and medical support in Relocation 2 (Tables 2 and 3). The consistent results across both relocations suggest the framework is reliable, adaptable, and sustainable for future use.

**Table 2:** Clinical and transport variables.

VARIABLES	Relocation 1(*n* = 16)	Relocation 2 (*n* = 17)
**Demographics**		
Female sex, *n* (%)	6 (37.5%)	3 (17.6%)
Male sex, *n* (%)	10 (62.5%)	14 (82.3%)
Median gestational age, weeks (IQR)	32.3 (24–39.6)	33 (23–41)
Median postmenstrual age, weeks (IQR)	39 (29–73.2)	40 (26.5–55.8)
Median birth weight, g (IQR)	1870 (730–3100)	1490 (860–2500)
Median weight at transport, g (IQR)	2700 (990–6030)	2420 (1520–3830)
**Clinical acuity**		
Infants on enteral feeds, *n* (%)	13 (81.2%)	13 (76.4%)
Median number of infusions (IQR)	1.5 (0–5)	2 (0–8)
Patients requiring >2 continuous infusions, *n* (%)	5 (31.2%)	8 (47.0%)
IV sedation/analgesia, *n* (%)	3 (18.8%)	7 (41.0%)
Patients receiving inotropic/vasoactive support, *n* (%)	1 (6.3%)	1 (5.8%)
Prostaglandin infusion, *n* (%)	2 (12.5%)	2 (11.7%)
Inhaled nitric oxide, *n* (%)	0 (0%)	1 (5.8%)
Central venous access (CVL/PICC/UVC), *n* (%)	8 (50.0%)	9 (53.0%)
Arterial lines (peripheral/UAC), *n* (%)	2 (12.5%)	1 (5.8%)
Intracardiac line, *n* (%)	0 (0%)	1 (5.8%)
Acute postoperative patients ( < 72 h), *n* (%)	0 (0%)	0 (0%)
**Respiratory support**		
No respiratory support, *n* (%)	6 (37.5%)	3 (17.6%)
Invasive ventilation, *n* (%)	2 (12.5%)	9 (52.9%)
Non-invasive ventilation (HFNC/CPAP/NIPPV), *n* (%)	8 (50.0%)	5 (29.4%)
• HFNC	1 (6.3%)	0 (0%)
• CPAP	4 (25.0%)	1 (5.9%)
• NIPPV	3 (18.8%)	4 (23.5%)
**Physiological stability and events**		
Hypothermia/hyperthermia	0 (0%)	0 (0%)
Equipment malfunction	0 (0%)	0 (0%)
Heart rate pre-transport, bpm (IQR)	147 (140.8–163)	151 (139–162)
Heart rate post-transport, bpm (IQR)	147 (132–159)	148 (138–159)
Temperature pre-transport, °C (IQR)	36.9 (36.78–37.0)	36.9 (36.8–37.0)
Temperature post-transport, °C (IQR)	36.7 (36.65–37.0)	36.9 (36.75–37.0)
Increase in FiO₂ during/post-transport, *n* (%)	5 (31.3%)	5 (29.4%)
Adverse events during transport	0 (0%)	0 (0%)
Interventions during transport	0 (0%)	0 (0%)
**Transport efficiency**		
Total relocation time, median (min)	225	197
Individual transport time, median (IQR), min	4 (4–5)	6 (5–7)

**Table 3:** Diagnostic and severity classification of transported neonates.

DIAGNOSIS	Relocation 1 (*n* = 16)	Relocation 2(*n* = 17)
**Complex Congenital Heart Disease**, *n* (%) • TGA (repaired), *n* • TGA (unrepaired, on prostaglandin/post-septostomy), *n* • HLHS (Post Norwood), *n* • HLHS (on prostaglandin/ASD stent and branch PA restrictors), *n* • Coarctation of Aorta (repaired), *n* • Large ASD/VSD, *n* • Congenital heart block (pacemaker in situ), *n*	**5 (31%)** 1 1 0 1 1 1 0	**4 (23%)** 0 1 1 1 0 0 1
**Bronchopulmonary dysplasia** [32], *n* (%) • Grade 1, *n* • Grade 2, *n* • Grade 3, *n*	**(13%)** 1 1 0	**3 (18%)** 0 1 2
**Necrotizing Enterocolitis,** *n* (%) • Medical, *n* • Surgical, *n*	**3 (19%)** 0 3	**4 (23%)** 2 2
**Neurological and metabolic disorders,** *n* (%) • Inborn errors of metabolism (Galactosemia, Mitochondrial), *n* • Brain abscess, *n* • Neurometabolic syndrome, *n* • Severe HIE with suspected neurometabolic disorder, *n*	**3 (19%)** 2 1 0 0	**2 (12%)** 0 0 1 1
**Other surgical conditions,** *n* (%) • Gastric perforation, *n* • Esophageal perforation, *n* • Pyriform aperture stenosis, *n*	**0 (0%)** 0 0 0	**3 (18%)** 1 1 1
**Other diagnosis,** *n* (%) • Patent ductus arteriosus (PDA), *n* • Severe hyperbilirubinemia (Rh Incompatibility on intensive phototherapy), *n* • Multiple congenital anomalies, *n* • Severe combined immunodeficiency disease (SCID), *n* • Prematurity (30 weeks), *n*	**3 (19%)** 0 1 0 1 1	**2 (12%)** 1 0 1 0 0

Detailed sub-classification is provided to reflect cohort heterogeneity and clinical complexity.

*TGA* Transposition of the Great Arteries, *HLHS* Hypoplastic left heart syndrome [single-ventricle physiology].

A previously published report of M-IHT during NICU renovation described the relocation of 16 neonates over a shorter distance (100 m over 90 min; mean transport time of 5.6 min per neonate). The cohort (mean gestation 35 weeks, mean birth weight 2259 g) was of lower acuity; most infants were self-ventilating, with one requiring hand ventilation due to the unavailability of a transport ventilator. Despite thorough preparation, the team reported physiological instability, equipment failures, and the need for post-transport stabilization. Parental involvement and effective communication were identified as important factors for a “smoother transport process” [13]. In contrast, our relocations involved higher-acuity neonates over a longer distance. Although our total operation time was longer, this reflected the deliberate 30-minute pacing interval designed to ensure safe sequencing. Individual transfer times were comparable to the previous publication [13], and overall efficiency improved in Relocation 2, suggesting system maturation with sustained efficiency and safety.

IHT involves multiple interdependent factors, including patient acuity, staffing, resources, transport route, destination readiness, time constraints, and family needs, all of which require robust coordination to ensure patient safety [1–6, 11–13, 19]. By viewing these elements as part of a larger interconnected system, we addressed them simultaneously during the planning, keeping safety, efficiency, and family-centered objectives in focus [14, 15]. Insights from the SAQ domains, such as teamwork and safety climate, guided the development of our standardized workflows and checklists, while visible and responsive leadership strengthened staff engagement. Job satisfaction, working conditions, and stress recognition were proactively managed through adequate, zone-based staffing, equipment readiness, intentional spacing of transfers, and clear and consistent communications [17]. OR/MS principles helped us identify operational constraints such as time limitations, staged room availability, transport sequencing, team composition, and equipment supply. By anticipating clinical (e.g., patient instability, care continuity), operational (e.g., equipment or process failures), and parental (e.g., anxiety or communication gaps) uncertainties [16], we aimed to prevent errors, minimize avoidable harm, and ensure coordination across people, processes, and environments, reflecting core patient-safety and resilience-science principles [17, 19].

Simulation-informed planning contributed to system resilience by informing time allocation and transfer sequencing. We allocated 30 min per neonate based on the estimated times for a full transport cycle, although individual transfers took only 4–6 min per infant. Bedside nurses accompanied the transport team, supporting both pre-transport preparation and post-transport care. This allowed simultaneous rather than sequential pre-transport preparations, creating natural pacing and buffer periods. These built-in safety margins enabled flexible sequencing, which, along with proactive leadership and pre-assigned standby teams, allowed unanticipated pre-transport clinical events and IT/infrastructure problems to be absorbed without disrupting the relocation. This adaptability reflects resilience-science and systems-thinking principles, in which systems are designed not only for efficiency under ideal conditions but also for reliability when challenges arise [8, 15].

Clear and structured communication was fundamental to the positive staff perceptions. In response to the feedback for earlier, broader communication, we disseminated the NICU Mass Transport Plan and role-specific documents well in advance. This improved staff engagement, supported frontline teams, and promoted consistency. A five-phased (week prior, day before, night before, relocation day, and post-relocation review) relocation task checklist informed the development of role-specific checklists, while the standardized patient data sheet ensured continuity of care by the receiving team. This aligns with evidence that checklists improve adherence to safety standards and reduce variability, important factors in error prevention [20–22]. Furthermore, staff participation in structured huddles supported coordination and shared situational awareness, promoting standardization and accountability. This supports literature that MDT safety briefings improve patient safety [23–25]. The ^**“**^Transport Huddle Sheets”, initiated the night before relocation, documented each neonate’s priority number, respiratory support, and room allocations. The estimated departure times, sequencing, and routes were finalized during the morning huddle, which, along with any overnight updates, allowed us to schedule low-risk and stable ventilated neonates first, and the high-acuity cases last to minimize disruption should instability occur. Shared across teams, Huddle sheets supported real-time adjustments and Vocera-based communication throughout the relocation, strengthening coordination and shared ownership. Together, these tools standardized communication, improved situational awareness, efficiency, and safety [26].

A proactive equipment management plan included pre-relocation testing, backup emergency carts at the receiving unit, and structured workflows for battery and gas management, cleaning, and equipment reuse. These measures supported real-time troubleshooting, infection prevention, and operational efficiency, consistent with findings that standardized processes, routine checks, and proactive maintenance enhance patient safety during IHT [26].

Effective leadership, attention to teamwork, and favorable working conditions are known to enhance staff engagement [27]. Consistent with this, senior nursing and medical staff were present throughout both relocations, providing oversight and real-time decision-making. Their presence strengthened team confidence, kept timelines on track, and supported team morale and patient safety.

Finally, proactive parental engagement through letters and face-to-face communications, including invitations to accompany infants, and updates before and soon after transfer, was reflected in positive parent survey findings. These strategies reflect Family Integrated Care principles intended to reduce anxiety, foster trust, and support continuity of care and bonding [5, 28, 29]. Feedback from one parent who was unable to accompany their neonate highlighted the need for timely updates through multiple communication channels.

The best-practice recommendations from the neonatal-transport literature, though largely focused on inter-hospital transport, emphasize thorough preparation and stabilization before transfer; dedicated and well-trained teams; structured communication tools, standardized protocols and checklists; parental engagement; and attention to environmental conditions and equipment readiness [2, 5, 6, 28–31]. Our experience confirmed the importance of these principles for IHT and demonstrated how structured planning can ensure safety during complex operations. This framework represents operational principles that are scalable and adaptable to other relocation scenarios involving different floors or buildings within a hospital campus. Such scenarios may introduce additional environmental and operational constraints. Vertical transport requires elevator use, and securing dedicated elevator access may reduce delays and operational disruption. Longer and variable transport routes during inter-building transfers, including ramps or outdoor pathways, as well as limitations in oxygen supply and battery capacity, must be proactively anticipated and mitigated. Elevator transfers or extended routes may temporarily isolate the transport team from the NICU’s immediate emergency support resources, requiring clear escalation pathways and rapid access to backup personnel and equipment if clinical deterioration occurs. These constraints may also prevent parents from accompanying the transport in restricted spaces (e.g., elevators), requiring alternative communication strategies to support parental reassurance and continuity of care. In such scenarios, context evaluation becomes particularly important. Simulation-informed planning, multidisciplinary team discussions, real-time observation, and the application of OR/MS principles can help identify system constraints and optimize resource allocation. Proactive adaptation of the work system to maintain safe and coordinated operations, including consideration of thermoregulation and infection control, aligns with the principles of resilience science.

### Limitations

This study has several limitations. It is a single-center experience at a quaternary NICU with a specific architectural layout and resource model, which may limit generalizability. The sample size was relatively small, and the evaluation of staff and parental perceptions relied on voluntary survey data. Response rates were approximated from staff known to be on duty; while this ensured the survey captured the views of those most directly involved, it introduces potential selection and response bias. Additionally, there was no simultaneous control group; however, the iterative comparison between the two relocation phases provided internal learning and process validation. Finally, although there were no adverse events, the small cohort was insufficient to detect rare safety events.

## Conclusion

M-IHT of critically ill infants can be conducted safely and efficiently using structured, system-based frameworks integrating people, processes, environment, and family-centered care. This project highlights the value of proactive simulation-informed planning, consistent communication, and visible decisive leadership in a complex sociotechnical operation. The ability to repeat the process with consistent outcomes highlights its reliability, adaptability, and sustainability for broader use. The structured use of safety tools, contextual adaptation, and systems thinking provides transferable lessons for other high-risk healthcare operations.

## Supplementary information

Appendix S1-S5

## Data Availability

All data generated or analyzed during this study are included in this article and its supplementary material files. Further inquiries can be directed to the corresponding author.

## References

[1] Delacrétaz R, Fischer Fumeaux CJ, Stadelmann C, Rodriguez Trejo A, Destaillats A, Giannoni E. Adverse events and associated factors during intrahospital transport of newborn neonates. J Pediatr. 2022;240:44–50. 10.1016/j.jpeds.2021.08.074. 34480917

[2] Haydar B, Baetzel A, Elliott A, MacEachern M, Tweedy K, Park PK. Adverse events during intrahospital transport of critically ill children: a systematic review. Anesth Analg. 2020;131:1135–45. 10.1213/ANE.0000000000004585. 32925334

[3] Murata M, Nakagawa N, Kawasaki T, Yasuda H, Ishihara S, Shigeoka H, et al. Adverse events during intrahospital transport of critically ill patients: a systematic review and meta-analysis. Am J Emerg Med. 2022;52:13–9. 10.1016/j.ajem.2021.11.021. 34861515

[4] Bastug O, Gunes T, Korkmaz L, Elmali F, Kucuk F, Ozturk MA, et al. An evaluation of intra-hospital transport outcomes from a tertiary neonatal intensive care unit. J Matern Fetal Neonatal Med. 2016;29:1993–8. 10.3109/14767058.2015.1072158. 26335382

[5] Lengauer C, Grass B. Neonatal transport: parental needs and potential for improvement—a single-center cross-sectional study. J Matern Fetal Neonatal Med. 2024;37:2356036. 10.1080/14767058.2024.2356036. 39584505

[6] Latzke M, Schiffinger M, Zellhofer D, Steyrer J. Soft factors, smooth transport? The role of safety climate and team processes in reducing adverse events during intrahospital transport in intensive care. Health Care Manag Rev. 2020;45:32–40. 10.1097/HMR.0000000000000188 29176495

[7] Carayon P, Xie A, Kianfar S. Human factors and ergonomics as a patient safety practice. BMJ Qual Saf. 2014;23:196–205. 10.1136/bmjqs-2013-001812 PMC3932984 23813211

[8] Association for Project Management. What is systems thinking? Association for project management. Available from: https://www.apm.org.uk/resources/find-a-resource/what-is-systems-thinking/.

[9] Carayon P, Schoofs Hundt A, Karsh BT, Gurses AP, Alvarado CJ, Smith M, et al. Work system design for patient safety: the SEIPS model. Qual Saf Health Care. 2006;15:i50–8. 10.1136/qshc.2005.015842. PMC2464868 17142610

[10] Holden RJ, Carayon P, Gurses AP, Hoonakker P, Hundt AS, Ozok AA, et al. SEIPS 2.0: a human factors framework for studying and improving the work of healthcare professionals and patients. Ergonomics. 2013;56:1669–86. 10.1080/00140139.2013.838643. PMC3835697 24088063

[11] Bergman L, Chaboyer W, Pettersson M, Edebol-Carlman U, Carlström E, Ringdal A. Development and initial psychometric testing of the Intrahospital Transport Safety Scale in intensive care. BMJ Open. 2020;10:e038424 10.1136/bmjopen-2020-038424. PMC7552847 33040010

[12] Li S, Hou S, Deng X, Chen S, Wang H, Tang L, et al. Reliability and validity assessment of the Chinese version of the Intrahospital Transport Safety Scale (IHTSS) in intensive care units. BMC Nurs. 2024;23:296. 10.1186/s12912-024-01906-z. PMC11057123 38684975

[13] Kumar R, Wasim S, Gupta G, Mathur S, Kahlon DS. Experience of mass intrahospital neonatal transport: impact on vitals. Cureus. 2022;14:e22785. 10.7759/cureus.22785. PMC8976189 35382196

[14] Ogrinc G, Davies L, Goodman D, Batalden P, Davidoff F, Stevens D. SQUIRE 2.0 (Standards for Quality Improvement Reporting Excellence): revised publication guidelines from a detailed consensus process. BMJ Qual Saf. 2016;25:986–92. 10.1136/bmjqs-2015-004411 PMC5256233 26369893

[15] Provan DJ, Woods DD, Dekker SWA, Rae AJ. Safety-II professionals: how resilience engineering can transform safety practice. Reliab Eng Syst Saf. 2020;195:106740. 10.1016/j.ress.2019.106740.

[16] Klein TL, Thielen C. Patient transport in hospitals: a literature review of operations research and management science methods. Oper Res Data Anal Logist. 2025;45:200472. 10.1016/j.ordal.2025.200472.

[17] Sexton JB, Helmreich RL, Neilands TB, Rowan K, Vella K, Boyden J, et al. The safety attitudes questionnaire: psychometric properties, benchmarking data, and emerging research. BMC Health Serv Res. 2006;6:44. 10.1186/1472-6963-6-44. PMC1481614 16584553

[18] Carter N, Bryant-Lukosius D, DiCenso A, Blythe J, Neville AJ. The use of triangulation in qualitative research. Oncol Nurs Forum. 2014;41:545–7. 10.1188/14.ONF.545-547. 25158659

[19] Kim L, Lyder CH, McNeese-Smith D, Leach LS, Needleman J. Defining attributes of patient safety through a concept analysis. J Adv Nurs. 2015;71:2490–503. 10.1111/jan.12715. 26122016

[20] Williams P, Karuppiah S, Greentree K, Darvall J. A checklist for intrahospital transport of critically ill patients improves compliance with transportation safety guidelines. Aust Crit Care. 2020;33:20–4. 10.1016/j.aucc.2019.02.004. 30981603

[21] Fanara B, Manzon C, Barbot O, Desmettre T, Capellier G. Recommendations for the intra-hospital transport of critically ill patients. Crit Care. 2010;14:R87. 10.1186/cc9018. PMC2911721 20470381

[22] Comeau OY, Armendariz-Batiste J, Woodby SA. Safety first! Using a checklist for intrafacility transport of adult intensive care patients. Crit Care Nurse. 2015;35:16–26. 10.4037/ccn2015991. 26427972

[23] Franklin BJ, Gandhi TK, Bates DW, Huancahuari N, Morris CA, Pearson M, et al. Impact of multidisciplinary team huddles on patient safety: a systematic review and proposed taxonomy. BMJ Qual Saf. 2020;29:1–2. 10.1136/bmjqs-2019-009911. 32265256

[24] Grabinski Z, Duncan E, Patel K, Shah A, Olinde A, Giannetti N, et al. Improving the safety of pediatric emergency departments for inpatient transfers of care. Pediatrics. 2025;156:e2024068541. 10.1542/peds.2024-068541. 40467066

[25] Lin SJ, Tsan CY, Su MY, Wu CL, Chen LC, Hsieh HJ, et al. Improving patient safety during intrahospital transportation of mechanically ventilated patients with critical illness. BMJ Open Qual. 2020;9:e000698 10.1136/bmjoq-2019-000698. PMC7202726 32317274

[26] Yang SH, Jerng JS, Chen LC, Huang SF, Chang LC, Lin CK, et al. Incidence of patient safety events and process-related human failures during intra-hospital transportation of patients: retrospective exploration from the institutional incident reporting system. BMJ Open. 2017;7:e017932 10.1136/bmjopen-2017-017932. PMC5695373 29101141

[27] Huang CH, Wu HH, Lee YC, Li X. The critical role of leadership in patient safety culture: a mediation analysis of management influence on safety factors. Risk Manag Health Policy. 2024;17:513–23. 10.2147/RMHP.S446651. PMC10929255 38476201

[28] Fidler HL, McGrath JM. Neonatal transport: the family perspective. Newborn Neonate Nurs Rev. 2009;9:187–90. 10.1053/j.nainr.2009.09.010.

[29] BLISS. Transfers of premature and sick babies. London: BLISS; 2015. Available from: https://s3.eu-west-2.amazonaws.com/files.bliss.org.uk/images/Transfers-of-premature-and-sick-babies.pdf

[30] European Standards of Care for Newborn Health Project Members. European Standards of Care for Newborn Health: Project Report; 2021. Available from: https://newborn-health-standards.org/wp-content/uploads/2023/04/ESCNH_Report_EN_web.pdf 10.1111/apa.15712 33290600

[31] Whyte HE, Jefferies AL. Canadian Paediatric Society, Fetus and Newborn Committee. The interfacility transport of critically ill newborns. Paediatr Child Health. 2015;20:265–75. PMC4472056 26175564

[32] Jensen EA, Dysart K, Gantz MG, McDonald S, Bamat NA, Keszler M, et al. The diagnosis of bronchopulmonary dysplasia in very preterm infants. An evidence-based approach. Am J Respir Crit Care Med. 2019;200:751–9. 10.1164/rccm.201812-2348OC PMC6775872 30995069

