# Supplementary material for: Mass intrahospital transport of the neonatal intensive care unit: a systems-based approach to safety, efficiency, and family-centered care
Source: J Perinatol. 2026 May 27;46(11):2179–87. doi: 10.1038/s41372-026-02721-x (PMC13731127; doi:10.1038/s41372-026-02721-x)

Supplementary Appendix S1

Relocation 2 Checklist (3A to 3C)

This timeline plan ensures a structured and coordinated approach to the mass transport of neonates. By adhering to this schedule, we aim to minimize disruptions, ensure the safety of the neonates, the well-being of NICU staff, and support for the parents. We strive to maintain clear communication and good coordination among all team members.

Senior leaders will provide overall strategic leadership and will initiate this checklist on **(Date)**

**A week before the Move**	1. The whole NICU staff was informed of the date<br>2. Teams identified and organized.<br>3. Core members meet on the **Date** to discuss the plan and its execution.<br>4. Updating the identified teams, both Medical & Nursing, with the documents, plan, and execution.<br>5. Ensure the 3 C, including the Clerk (IT dock) and nursing station, doctors (including attending), nursing offices, and RT offices, are fully prepared for the move.<br>6. Facilities informed about the transfer date to ensure Infection control cleaning, Electric, Facilities, IT, Biomed, Equipment, Porters, Security, Pharmacy (Omnicell), and supplies, and inform them of the day of the move, and to stand by.<br>7. To reconfirm that all the Neonatal staff access badges work in 3 C, including BMEs<br>8. Confirm with BioMed to ensure at least a few rooms are set in 3C to receive babies at 0830 am on the **Date**. The remaining rooms can be set as we go on **Date**<br>9. All the parents are informed about the plan and date.<br>10. Route planned and teams informed.<br>11. All the potential elective procedures/investigations, including ROP, were identified and cancelled.<br>12. OBGY informed to cancel any elective high-risk deliveries.<br>13. All the subspecialties (including CICU/PICU) and Networks were informed about the move date and plan<br>14. Final Teams Meeting with all the teams involved on the **DATE** to discuss the plan collectively, address any questions, and finalize the details.<br>15. Arrange 2 flip chart easels for 3 A entry banners and store them in the family room.	☐<br>☐<br>☐<br>☐<br>☐<br>☐<br>☐<br>☐<br>☐<br>☐<br>☐<br>☐<br>☐<br>☐<br>☐
**A Day Before the Move**	1. Identify HDU babies in NICU and transfer them to HDU unit.<br>2. Reconfirm with BioMed to ensure at least a few rooms are set in 3 C to receive babies at 0830 am on the **Date**. The remaining rooms can be set as we go on **Date**<br>3. Reconfirm with OBGY to cancel any high-risk elective sections/deliveries.<br>4. A reminder to all the subspecialties about the Move.<br>5. Quick catch up with all the involved Team in the afternoon and reconfirm the plans<br>6. Recheck documentation files, including A1-size route banners in 3A in place and for each 3C entry door for distribution to the night team and the next-day team members involved in the move.	☐<br>☐<br>☐<br>☐<br>☐<br>☐

Supplementary Appendix S1

Relocation 2 Checklist (3A to 3C)

**A Night before the Move**	1. **The Patient sheets** are to be given to the bedside nurse for initiation.<br>2. **Transport Huddle sheets** are initiated during the ward rounds.<br>3. **The priority numbers** suggested/allocated to the babies for discussion in the Morning Transport huddle. *(First transfer the Stable ventilated babies, followed by the Sick ventilated babies, then start with the non-ventilated babies)*<br>4. **The Priority number 1 babies for the first transfer at 0830,** confirm with the bedside nurse to prepare **by 08:15**.<br>5. **Visit and inspect 3 C** and ensure all is OK.<br>6. **Assign the room numbers** and **entry door numbers** to the babies on the transport huddle sheet.<br>7. **Route banners:** are put on the door/wall and corridors as appropriate.<br>8. **Entry banners** are put at the turn where the main hospital corridor joins the 3 A entry corridor<br>9. **The main Route Map** is put on a flipchart easel for reference in the front of clerk's desk in the 3 A.<br>10. **14 photocopies** of each of the initiated Red/Blue Transport huddle sheets after completion for distribution to the morning teams.<br>11. **Print the extra NICU handover sheets** for the morning handover for the 3 C Teams and both the Transport teams<br>12. **Print extra CNL Handover sheets** for the receiving units CNLs.	☐<br>☐<br>☐<br><br><br>☐<br><br>☐<br>☐<br><br>☐<br>☐<br><br>☐<br><br>☐<br><br>☐<br><br>☐

MOVE DAY: ***Parents are allowed to accompany the baby during the transfer if they are present during the morning rounds and wish to.***

**0700:**	NICU Handover in the conference room is attended by all members, including the both 3 C and Transport teams.	☐
**0730**	NDTT huddle as usual for routine planning and ensure there are no elective sections that may need admission or any planned procedures for the day.	☐
**0730 to 0800**:	**Red/Blue Transport Huddle**: **(Led by respective Transport teams)**<br>➢ Each Transport teams, sending teams, and 3 C Teams hold separate Transport huddles in the consultant room and the conference room simultaneously **using Transport huddle sheets.**<br>➢ Address any acute issues that have arisen overnight with any of the babies and discuss the transport plan for that baby.<br>➢ Identify any Sick baby on HFOV or Nitric oxide and discuss any issues and their transport plan as needed.<br>➢ Discuss the Transport huddle sheets (Confirm baby's priority numbers, Allocated NICU room numbers, EXIT Route and entry door numbers, and estimated departure time. (Starting at 08:30, followed by 30 mins between babies) ***(Stable ventilated, then the Sick ventilated, then non-ventilated)***<br>➢ Discuss contingency plan in case of emergency on the route: NDTT is called to take care of the acute illness and stabilization, the baby will go to the procedure room/its original room in the 3 A (unless very close to 3 C)<br>➢ Allow parents (if they are present) to accompany the baby during the transfer.	☐<br>☐<br>☐<br>☐<br>☐<br>☐

Supplementary Appendix S1

Relocation 2 Checklist (3A to 3C)

0800-0830:

Quick TEAM huddle in the NICU conference room as usual ***(Quick and brief)*** ☐

3 A TEAMS:

1. The red and blue team starts rounds. ☐
2. They see the babies based on their priority number. **(Refer to their copy of the respective transport huddle sheet.)** ☐
3. They confirm the priority number, room number, the entry door number, and the estimated departure time to the bedside nurse. ☐
4. They enter the day management plan for the baby in the patient sheet. ☐
5. They continue their ward rounds as per the priority number of the babies. ☐

3 C TEAMS:

1. Heads to 3 C. ☐
2. Ensure the Banners on the corridor entrance are displayed properly. ☐
3. Ensure rooms allotted to the 1ST incoming prioritized babies are ready. ☐
4. Ensure the doors of the rooms are opened and Monitors set up. ☐
5. Confirm last-minute readiness with the CNLs and address any issues as needed. ☐

Both TRANSPORT TEAMS: **(This is Extra time for troubleshooting)**

1. Catch up with their respective team members (Middle grade, RT, and Attending). Acknowledge the plan as discussed in the transport huddle for the day, as per the Red/Blue Transport sheet (baby's priority numbers, Allocated room numbers, EXIT Route, and entry door numbers, and estimated departure time. (Starting at 08:30, followed by 30 mins between babies) ***(Stable ventilated, the Sick ventilated, then non-ventilated OR as per the need)*** ☐
2. Address any acute issues that have arisen overnight with any of the babies and discuss the transport plan for that baby. ☐
3. Identify any Sick babies on HFOV or Nitric oxide and discuss any issues and transport plan. (Need for an extra RT: NDTT RT will help) ☐
4. Role allocation for monitoring during transport. ☐
5. Discuss contingency plan in case of emergency on the route: NDTT is called to take care of the acute illness and stabilization, the baby will go to the procedure room in 3 A (unless very close to 3 C) ☐
6. Shuttle batteries run out in 1 hour, so keep them plugged in during pretransfer and post transfer (allocate a person) ☐

Oversight Neonatal specialists (Stationed in both NICUs)

1. Receive/send babies and maintain the oversight of operations. ☐
2. Check if the route banners are properly displayed ☐
3. Remind the bedside nurse to ensure the transport monitors are properly associated and dissociated to prevent losing vital information ☐
4. Ensure the patient location is changed on the Cerner. (Remind the clerks) ☐
5. Ensure patient sheets are complete and are uploaded to Cerner. ☐
6. Remind the nurses to call parents and update them after the transfer. ☐
7. Collect all the Patient Sheets and all the file folders from all the involved teams after completing the MOVE. ☐
8. Hand over the documents to Oversight Neonatal attending to ensure proper disposal of the documents for privacy and confidentiality reasons. ☐

Supplementary Appendix S1

Relocation 2 Checklist (3A to 3C)

	9. Ensure the Staff survey gets disseminated after completing the Move.<br>10. Ensure the parents' survey gets disseminated the day after the Move.<br><br>**OVERSIGHT CNL:**<br>1. Confirm with the bedside nurses about the patient sheet, priority number, 3C allocated room number, 3C entry door number, and the estimated departure time.<br>2. Confirms with the bedside nurse to inform the parents that morning and document the time on the patient sheet.<br>3. Remind the bedside nurse to ensure the transport monitors are properly associated and dissociated to prevent losing vital information.<br>4. Remind the nurses to upload the patient sheet onto Cerner after the transfer.<br>5. Remind the nurses to call parents and update them after the transfer.<br>6. Ensure the Staff survey gets disseminated to the nurses and encourage them to complete it after the Move.<br>7. Ensure the parents' survey gets disseminated the day after the Move.	☐<br>☐<br>☐<br>☐<br>☐<br>☐<br>☐<br>☐<br>☐
0830 - 1130:	1. Start the transfers with ventilated babies at 0830, transferring simultaneously one for each team based on the priority number.<br>**ALLOW PARENTS IF THEY ARE PRESENT AND WISH TO ACCOMPANY THE BABY DURING THE TRANSFER.**<br>2. **BIOMED** will start setting up the remaining rooms in receiving unit.<br>3. **Shuttle batteries** run out in 1 hour, so keep them plugged in during pretransfer and as soon as the baby reach in 3 C (**allocated person**)<br>4. On return, PCP cleans and then prepares for the next baby.<br>5. Transport RT will exchange the "dirty" machine for a "clean" machine at the RT Storeroom on the way back to 3 A (Role of RT Equipment Coordinator)	☐<br>☐<br>☐<br>☐<br>☐
11:30 – 12:30	**Mid-Review and Adjustments** to the plan **(if needed)**<br>**Break: REFRESHMENTS IN THE 3C CONFERENCE ROOM.**	☐
12:30 – 14:30	Transfer of Remaining Babies as per the plan.	☐
14:30 – 1500 (or later)	1. Ensure all WhatsApp boards are moved to the respective rooms in 3 C<br>2. Ensure ANNP/NH office move all the posters and other furniture etc.<br>3. Facilities informed and confirmed the transfer and requested to transfer any leftover equipment after the move in the evening.	☐<br>☐<br>☐
15:00 - 1600 (or earlier)	**Post-MOVE Review:**<br>1. All the teams gather, discuss, and conduct a thorough review of the transfer process and a debrief in the NICU conference room.<br>2. Re-ensure all the documents are complete and collected (handover to Oversight neonatal consultant)<br>3. Re-ensure parents are updated post-transfer.<br>4. Re-ensure all the patient sheets get uploaded to their respective Cerner<br>5. Communicate Relocation completion and final updates to stakeholders (subspecialties, hospital leadership, network, etc.)	☐<br>☐<br>☐<br>☐<br>☐<br>☐

Supplementary Appendix S1

Relocation 2 Checklist (3A to 3C)

	6. Distribute the staff and Patient survey to all the involved and socialize in the operation group and encourage all to complete.	
1600 onwards	1. Porters/facility to move all the remaining NICU equipment.<br>2. Final inspection for the 3 A.	☐<br>☐
1900	Night handover in the NICU conference room.	☐
**After the Move**	Inspection and Handover of 3 A to the operations	☐

Supplementary Appendix S2
Workforce & Equipment Management (Relocation 2: 3A to 3C)

Senior leaders: Strategic leadership
Oversight Neonatal consultant/CNL: Oversight of the overall operation.
Zone-based Neonatal Specialist: Oversight of the operations in their zone, ensure good communication and smooth coordination, and ensure all the documentation is appropriately completed and collected. Parents will be informed and updated, and a post-move survey will be distributed.
RT lead: will oversee the RT operations.
Biomed Lead: oversee the Biomed operations.

NIGHT ON-CALL TEAM BEFORE MOVE: Neonatal Consultant+ Neonatal specialist+ ANNP;

Primary NICU DAY TEAMS:

1. **Red team: Neonatal Consultant,** middle grade, CNL, RT
2. **Blue team**: **Neonatal Consultant,** middle grade, CNL, RT

} **Oversight Neonatal Specialist**

GREEN (HDU) TEAM: Neonatal Consultant, middle grade (Postnatal + Neonatal delivery team (NDT))

NDTT TEAM: Neonatal Consultant, middle grade (Postnatal + NDT) + NDT CNL+ RT (may also help as an extra pair of hands to move the babies on HFOV/Nitric)

3A MOVE TEAMS: **(Primary NICU teams)**

1. **RED Move team: Neonatal Consultant,** middle grade, CNL, RT
2. **Blue Move team: Neonatal Consultant,** middle grade, CNL, RT
3. **3A 2 PCP**
4. **Pharmacist**

3C TEAMS: (Temporary teams stationed to receive babies)

1. **3C Red team**: **Neonatal Consultant,** middle grade, CNL, RT
2. **3C Blue team**: **Neonatal Consultant,** middle grade, CNL, RT
3. **3C 2 PCP**
4. **Pharmacist**

} **Oversight Neonatal Specialist** (3C Red and Blue teams)

NIGHT TEAMS: (Move day)

1. **Green (HDU) Night team**: **Neonatal consultant (coming at 1500)** + middle grade.
2. **Red/Blue Night**: **Neonatal Consultant** + 2 Middle grade

NURSING TEAM:

1. **Float CNs for the day**: **4 CNs** (1 NDT CN 3 FLOAT): 1 is for the regular NDT team, and other 3 are to help around in 3A, and after a significant number of babies get transferred, they needed in 3C
2. **Extra Nursing staff: 5 (1 CNs +4CN's):** (2 MOVE nurses + 3 will be stationed in 3C (1 red and blue team each + 1 on 3A nursing station)

RT TEAM:

Regular 3 A: 3 RTs (including 1 NDT). 1 of these will move to 3C when the majority of patients have moved.

1. **Move team RTs**: **2 RTs** dedicated to the 2 Move teams.

Supplementary Appendix S2

Workforce & Equipment Management (Relocation 2: 3A to 3C)

2. **Runner RT:** **1 extra RT** to move the existing ventilator to 3C and set it up in the patient room.
3. **3C RTs:** **2 RTs** for each team stationed in 3C to receive patients.

ANCILLARY TEAMS:

1. **Porters 4** (2 for Biomed and 2 for NICU)
2. **Biomed** team: **3** teams.
3. **Security guards 2** (1 each to guard 3A AND 3C doors: Ensures no obstruction and Corridors are clear to smooth transfers and coordinates the To and Fro movement from 3A AND 3C)

TEAMS FOLDERS:

A night before Move day, RED/BLUE TEAM FOLDER: night checklist, Red & Blue transport huddle sheet, **Patient sheets,** Mass transport plan, Route maps.

3C RED/BLUE TEAM FOLDERS: 3C RED/BLUE Team checklist, Photocopy of the Red transport huddle sheet, Mass transport plan, NICU handover sheet of the day, Route map.

3A RED TEAM FOLDERS: 3A Team checklist, Photocopy of the Red & Blue transport huddle sheet, Mass transport plan, NICU handover sheet of the day, Route map.

RED/BLUE TRANSPORT TEAM FOLDER: Transport team huddle checklist, Transport huddle sheets, Mass transport plan, NICU handover sheet of the day, Route map.

RT LEAD: RT coordinator checklist, Transport huddle sheets, Mass transport plan, Route map

Neonatal consultant (Lead): TIMELINE DOCUMENT CHECKLIST, Copy of all the Red AND Blue transport huddle sheet, Mass transport plan, NICU handover sheet of the day, Route map, and workforce document.

Neonatal Specialist (3C): Role-specific checklist, Copy of 3C Teams checklist, Photocopies of All Red & Blue transport huddle sheets, Mass transport plan, NICU handover sheet of the day, Route map, Staff Survey QR code, and Patient survey QR code.

Neonatal Specialist (3A): Role-specific Checklist, Copy of 3A Teams checklist, Photocopies of All Red & Blue transport huddle sheet, Mass transport plan, NICU handover sheet of the day, Route map, Staff Survey QR code, and Patient survey QR code

CNL (Lead): Role-specific Checklist, Timeline document checklist**,** Copy of Red AND Blue transport huddle sheet, Mass transport plan, CNL NICU Handover sheet, Route map, Staff Survey QR code, and Patient survey QR code.

CNM: Role-specific checklist, Timeline document checklist, Copy of Red AND Blue transport huddle sheet, Mass transport plan, CNL NICU handover sheet of the day, Route map, and workforce document.

CNM: Timeline document checklist, Mass transport plan, Route map, and workforce document

Senior leadership: Timeline document checklist, Mass transport plan, Route map, and workforce document.

Supplementary Appendix S2

Workforce & Equipment Management (Relocation 2: 3A to 3C)

EQUIPMENT Management:

- **BIOMED** will start setting up the remaining rooms in 3C after the 1st transfers.
- **4 Transport ventilators** dedicated to the move. **Another 2** are used for changing over after cleaning.
  - Transport RT will exchange the "dirty" machine for a "clean" machine at the RT Storeroom at B tower on the way back to 3A.
  - RT Equipment Coordinator is responsible for cleaning and equipment turnaround.
- **4 shuttles for the move**: Their batteries run out in 1 hour, so important to keep them plugged in during pretransfer and as soon as you reach there in 3C (dedicated person)
  - On return, PCP in 3C can quickly clean and then prepare for the next baby (need a dedicated person)
- For Sick babies on HFOV/Nitric oxide: Use the baby's own ventilator. Need an extra RT to help with this. (NDTT RT will help)
- Babies being transferred on their own ventilator will need Air along with O2 cylinders (To ensure enough air and O2 supply, and AIR/O2 pipes need to be plugged to the wall socket pretransfer and as soon as you reach 3C. **(Please instruct the RT'S)**

Supplementary Appendix S3

TRANSPORT HUDDLE SHEET (Relocation 2: 3A to 3C)

Initiated by the Night team on DATE and reconfirmed during the TRANSPORT huddle on the DATE morning.

For babies on HFOV or Nitric oxide: use the baby's own ventilator (Need an extra RT, NDTT RT will help)

Any other ventilation, including non-invasive: use Transport ventilator.

Priority numbers: Start with stable ventilated babies, the Sickest baby to be transferred last.

The move starts at 0830, and the Estimated Departure time is calculated based on priority number, and the average move time for babies is 30 minutes. (Sickest baby on nitric may take up to 30-40 mins)

Baby sticker (ID/MRN)	3A Room	Respiratory support	Nitric	Transport or Baby's Ventilator	Priority no.	Estimate Depart time	3C Room	3C entry door

Supplementary Appendix S4
Patient Data Sheet

BABY'S STICKER

Initiated by the bedside nurse during the night on DATE.
Priority number, ETD, 3C room number, and route 3C entry point will be conveyed to the nurse by the medical team.
PLEASE CONFIRM THIS INFORMATION ON THE NIGHT WARD ROUNDS
ENSURE BABY IS READY TO MOVE 15 MINUTES BEFORE THE estimated departure time.

3A Room: **Priority No:** **Estimated departure time:** **3C Room:** **3C entry (1st/2nd door):**

Parents aware: Yes/no

Infusions:

SERIAL #.	Name of the infusion	Rate

Feeding (Amount and time of last feeding): **Sedation type and dose:**

Respiratory support: SV ☐ LF ☐ HFNC ☐ NCPAP ☐ NIV ☐ Conventional/VG ☐ HFOV ☐ **iNo** ☐

PRE-TRANSFER VITALS: (Please complete at the time of Move (+/-10 mins)

TIME	TEMP	HR	RR	SPO2	BP	Blood Sugar	Respiratory Settings	iNO	FIO2	BLOOD GAS (Morning Gas or As indicated)
										Ph__ PCo2__<br>BE__ HCO3__

Neurology: Irritable ☐ Sedated ☐ Sleeping ☐ Muscle relaxed ☐ Pain score (if applicable):

TIME LEFT 3A:

DURING TRANSFER:

**RR (stable/Apnea/tachypnea),** mention Peak/lowest value		Peak value:	Lowest value:
**Spo2**: (Stable/desaturations), mention lowest SPO2		Peak value:	Lowest value:
**Fio2** (increase/decrease), mention PEAK value		Peak value:	
**HR** (Stable/Tachy/Brady), mention Peak/lowest value		Peak value:	Lowest value:
**Any instability/adverse events:**			
**Any intervention/treatment given during transport:**			

Supplementary Appendix S4
Patient Data Sheet

BABY'S STICKER

Initiated by the bedside nurse during the night on DATE.
Priority number, ETD, 3C room number, and route 3C entry point will be conveyed to the nurse by the medical team.
PLEASE CONFIRM THIS INFORMATION ON THE NIGHT WARD ROUNDS
ENSURE BABY IS READY TO MOVE 15 MINUTES BEFORE THE estimated departure time.

TIME ARRIVED IN 3C:

POST-TRANSFER VITALS: (please complete on arrival (+/-10 mins)

TIME	TEMP	HR	RR	SPO2	BP	Blood Sugar	Respiratory Settings	iNO	FIO2	BLOOD GAS (For all ventilated babies)
										Ph__ PCo2__<br>BE__ HCO3__

Neurology: Irritable ☐ Sedated ☐ Sleeping ☐ Muscle relaxed ☐ Pain score (if applicable):

Parents informed, and Time of call:

Ensure no patient belongings are left behind:

**Plan during the Morning rounds**: *(Ensure that this is completed during the morning rounds by the medical team)*

Vital trends during transfer, including range and mean of vitals and histogram if available.	**HR:**<br>**RR:**	**SPO2:**<br>**FIO2:**

Supplementary Appendix S5

ROUTE MAP: RELOCATION 2: 3A TO 3C

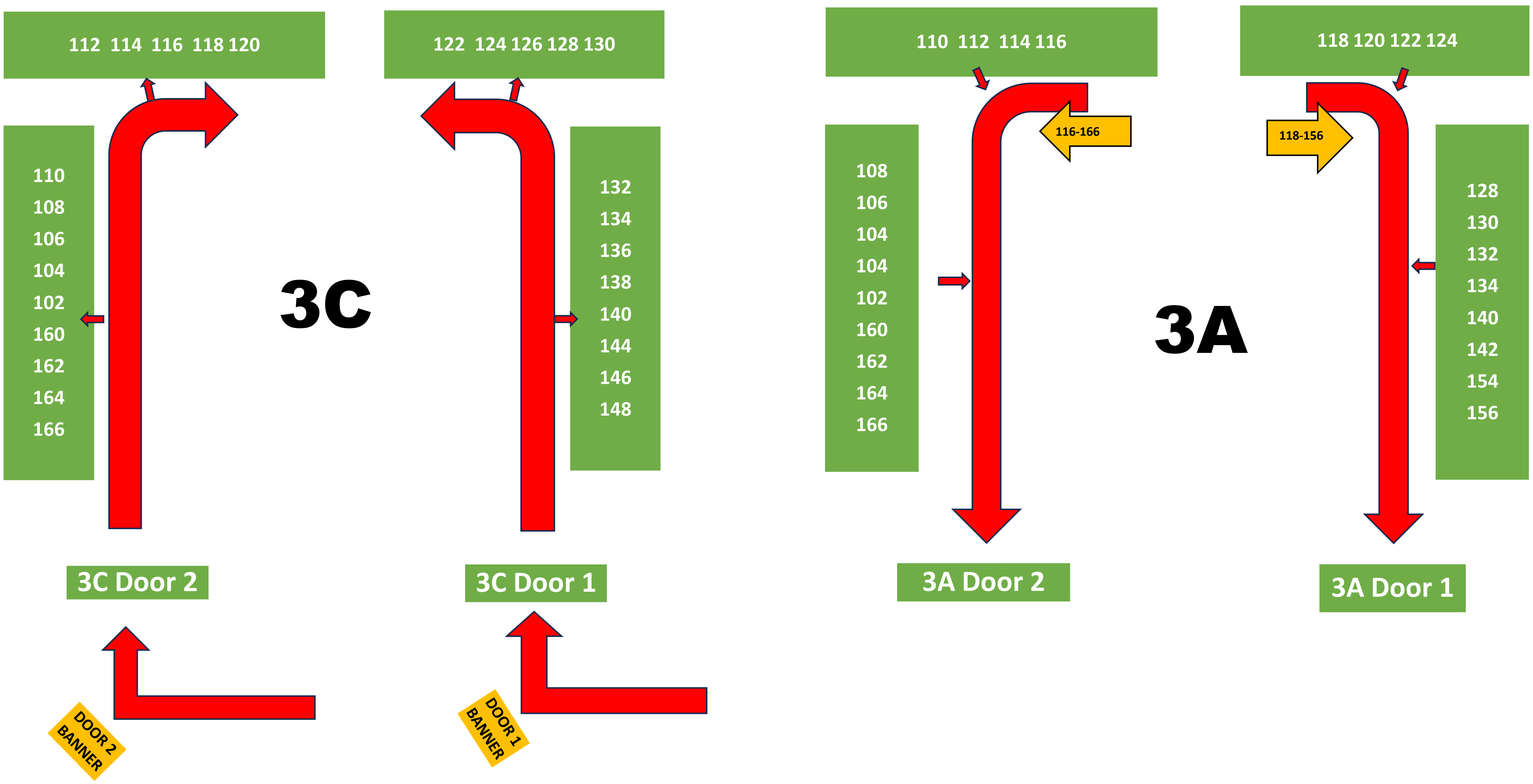

Supplement: Supplementary file 1 [file 41372_2026_2721_MOESM1_ESM.pdf]
